# Association of Finerenone Dose and Estimated Glomerular Filtration Rate (eGFR) Slope Changes in Patients With Diabetic Kidney Disease: A Real-World Retrospective Study

**DOI:** 10.7759/cureus.116611

**Published:** 2026-09-20

**Authors:** Hazuki Otani, Soichi Kurioka

**Affiliations:** 1 Department of Diabetology, Komatsu Hospital, Neyagawa-shi, JPN

**Keywords:** diabetic kidney disease (dkd), egfr slope, finerenone, type 2 diabetes mellitus, urinary albumin-to-creatinine ratio

## Abstract

Background

Finerenone demonstrates renoprotective effects in clinical trials; however, real-world evidence regarding the association between maintenance doses (10 mg vs. 20 mg) and estimated glomerular filtration rate (eGFR) slope changes remains scarce. We investigated the real-world impact of finerenone dosage and baseline clinical factors on eGFR slope trajectory in patients with diabetic kidney disease (DKD).

Methods

We retrospectively analyzed 104 patients with DKD who initiated finerenone and completed at least six months of follow-up. The primary outcome was the change in annual eGFR slope from the one-year pre-initiation period to the six-month post-initiation period. Secondary outcomes included changes in urinary albumin-to-creatinine ratio (UACR), urinary protein-to-creatinine ratio (UPCR), serum potassium, and blood pressure. Multivariable linear regression analysis was conducted to identify independent baseline factors associated with eGFR slope improvement.

Results

Following finerenone initiation, the overall eGFR slope improved from -5.20 to +0.27 mL/min/1.73 m²/year (p = 0.0144). Among subgroup analyses, significant slope improvement was observed in patients maintained on the 10 mg/day dose (n = 52, from -7.11 to +3.35 mL/min/1.73 m²/year, p = 0.00041), whereas the 20 mg/day group (n = 52) remained in a stable trajectory (-3.29 to -2.85 mL/min/1.73 m²/year, p = 0.865). In multivariable analysis, concomitant sodium-glucose cotransporter 2 (SGLT2) inhibitor use (B = +9.79, p = 0.016), younger age, lower baseline eGFR, and lower body mass index were independently associated with slope improvement. UACR decreased significantly (p = 0.0098), while serum potassium increased modestly (+0.086 mmol/L, p = 0.045) without clinically meaningful blood pressure changes.

Conclusions

Finerenone significantly attenuated eGFR decline in real-world patients with DKD, with slope improvement observed in patients maintained on the 10 mg/day dose. Concomitant SGLT2 inhibitor use was independently associated with greater improvement in eGFR slope, supporting the clinical utility and practical dose management of finerenone in routine practice.

## Introduction

Diabetic kidney disease (DKD) remains a major leading cause of end-stage kidney disease (ESKD) and cardiovascular events worldwide [1,2]. Over the past decade, the introduction of sodium-glucose cotransporter 2 (SGLT2) inhibitors alongside renin-angiotensin system (RAS) inhibitors has revolutionized the management of chronic kidney disease (CKD) in patients with type 2 diabetes mellitus (T2DM) [3]. However, substantial residual cardiorenal risk persists, highlighting an urgent unmet need for add-on disease-modifying therapies. 

Finerenone, a novel nonsteroidal mineralocorticoid receptor antagonist (MRA), selectively inhibits mineralocorticoid receptor overactivation, thereby mitigating renal inflammation and fibrosis [4]. Given the multifactorial pathophysiology of DKD involving both hemodynamic burden and chronic inflammation, MRAs have emerged as an essential therapeutic cornerstone [5,6]. Pivotal phase III clinical trials (FIDELIO-DKD and FIGARO-DKD) have demonstrated that finerenone significantly reduces urinary albumin-to-creatinine ratio (UACR) and slows estimated glomerular filtration rate (eGFR) decline, leading to a lower risk of renal and cardiovascular composite outcomes [7,8]. Following these landmark trials, several real-world studies from Japan have recently reported that finerenone attenuates the rate of eGFR decline (eGFR slope) in routine clinical practice [9-12].

In clinical trials, the dose of finerenone was routinely uptitrated from 10 mg to the maximum tolerated dose of 20 mg once daily based on eGFR and serum potassium levels [7]. However, in real-world practice, a substantial proportion of patients are maintained at 10 mg/day due to impaired baseline renal function or concerns regarding hyperkalemia. While the standard target dose of 20 mg/day is known to yield greater UACR reductions [10,13], the extent to which different finerenone doses (10 mg/day as a lower maintenance dose vs. 20 mg/day as a standard dose) and overall drug exposure impact long-term eGFR slope preservation remains poorly defined in real-world settings. 

To address this knowledge gap, we conducted a retrospective observational study evaluating the real-world associations between finerenone treatment and eGFR slope trajectories in Japanese patients with T2DM and DKD. Specifically, given that dose uptitration is frequently constrained in routine practice, we aimed to evaluate whether a lower maintenance dose (10 mg/day), as well as the standard dose (20 mg/day) and time-weighted average (TWA) dose, are associated with eGFR slope attenuation, rather than establishing dose superiority or causality. This investigation provides practical insights into routine dose management for preserving kidney function.

## Materials and methods

Participants

We retrospectively screened 175 patients with DKD who received finerenone (10 mg or 20 mg/day) at Komatsu Hospital between January 1, 2026, and March 31, 2026. Diabetic kidney disease was defined as an eGFR < 60 mL/min/1.73 m² and/or persistent albuminuria (UACR ≥ 30 mg/gCr or positive dipstick urinary protein) present for at least 3 months in individuals with type 2 diabetes mellitus. Patients who had initiated finerenone at other institutions (n = 3) were excluded. Additional exclusion criteria comprised: active non-diabetic primary renal diseases, acute kidney injury within three months prior to initiation, baseline serum potassium > 4.8 mmol/L precluding prescription initiation per the Japanese package insert, and incomplete medical records lacking baseline clinical parameters. Among the remaining eligible patients, 157 had at least one recorded clinical assessment both before and after finerenone initiation. Of these 157 patients, 53 patients who had initiated finerenone less than six months prior to the end of the study period were administratively censored without six-month follow-up data. Consequently, 104 patients who had been initiated on finerenone at least six months prior to the end of the study period and were continuously followed up for six months without discontinuation were defined as completers and included in the primary longitudinal trajectory analyses. A flow chart of the detailed patient selection process is illustrated in Figure 1. This study was approved by the Ethics Committee of Komatsu Hospital (approval No. 01-2026, approved on July 1, 2026) and was conducted in accordance with the Declaration of Helsinki (1975, as revised in 2013). Because of the retrospective and observational nature of the study, the requirement for written informed consent was waived by the Ethics Committee, and an opt-out approach was applied to guarantee patients the opportunity to decline participation.

**Figure 1:**
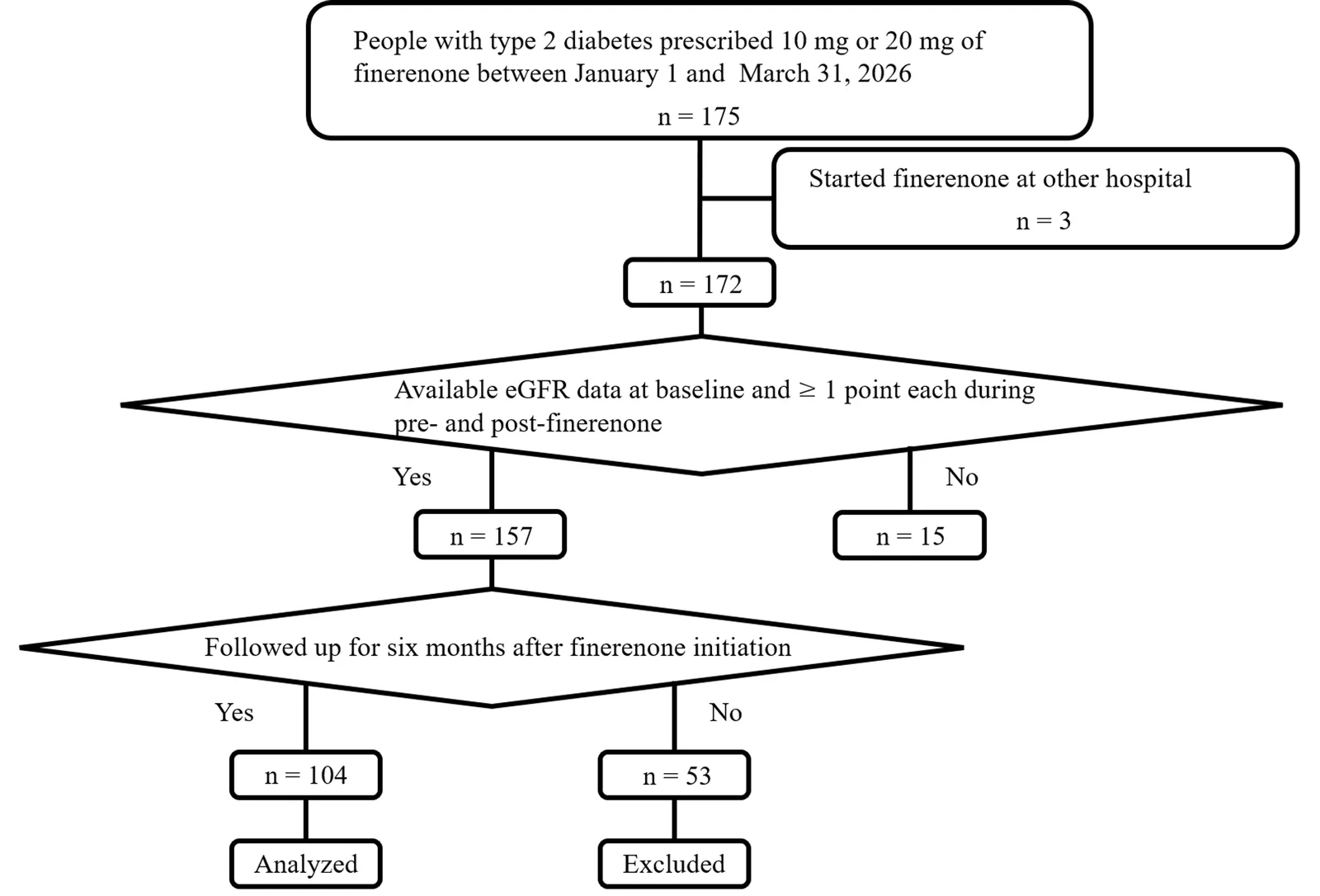
Flowchart of the patient selection process. A total of 175 patients with type 2 diabetes mellitus who were prescribed finerenone were initially screened. Three patients were excluded because finerenone was initiated at another hospital. Baseline and at least one pre- and post-initiation eGFR measurement were available for 157 patients. Among these, 104 patients completed a six-month follow-up after finerenone initiation and were included in the final analysis. eGFR, estimated glomerular filtration rate.

Outcome measure

The primary outcome was the change in the estimated glomerular filtration rate (eGFR) slope between the pre-finerenone period and the post-finerenone period. Serum creatinine-based eGFR values were evaluated at 12 and 6 months prior to finerenone initiation, at baseline (Month 0), and at 1, 3, and 6 months post-initiation. Secondary outcomes included changes in the urinary albumin-to-creatinine ratio (UACR) and urinary protein-to-creatinine ratio (UPCR). Safety outcomes included changes in serum potassium levels, as well as systolic and diastolic blood pressure.

Subgroup analyses for eGFR slope changes were performed based on baseline eGFR categories representing CKD stages: stage G1-G2 ( 60 mL/min/1.73 m^2^), stage G3a (45 to < 60 mL/min/1.73 m^2^), stage G3b (30 to < 45 mL/min/1.73 m^2^), and stage G4 (< 30 mL/min/1.73 m^2^), as well as maintenance finerenone doses (the standard 20 mg/day dose vs. the lower 10 mg/day maintenance dose). To identify independent predictors of eGFR slope improvement (Δslope), baseline clinical variables-including age, sex, body mass index (BMI), baseline eGFR, and concomitant use of SGLT2 inhibitors and glucagon-like peptide-1 (GLP-1) receptor agonists-were evaluated. Patients receiving concomitant SGLT2 inhibitors had been maintained on a stable dose prior to finerenone initiation, and no patients initiated, adjusted, or discontinued SGLT2 inhibitors during the six-month observation period.

To account for dose titrations during the observation period, the time-weighted average (TWA) finerenone dose (mg/day) over six months was calculated as follows:

TWA dose (mg/day) = ((10 × days on 10 mg) + (20 × days on 20 mg)) / total observation days

Patients were stratified into two groups based on a threshold of 15 mg/day: TWA < 15 mg/day and TWA ≥15 mg/day.

Statistical analysis

All statistical analyses were performed using EZR version 1.68 (Saitama Medical Center, Jichi Medical University, Saitama, Japan). Continuous variables are expressed as mean ± standard deviation (SD) for normally distributed data, or as median [interquartile range (IQR)] for non-normally distributed data. Categorical variables are presented as frequencies and percentages (n, %). For comparisons of baseline characteristics between two independent groups (10 mg vs. 20 mg maintenance dose groups, and TWA < 15 mg/day vs. TWA ≥ 15 mg/day groups), the unpaired Student's t-test or Welch's t-test was used for normally distributed continuous variables, the Mann-Whitney U test was used for non-normally distributed continuous variables (e.g., UACR), and the chi-square test or Fisher's exact test was used for categorical variables. While linear approximation models are commonly used in large-scale clinical trials, we calculated annual eGFR slopes using a direct difference-based model to evaluate the pure trajectory of kidney function decline, accounting for the relatively short six-month post-treatment period and missing intermediate data points. Specifically, the pre-treatment annual eGFR slope was calculated as the change from 12 months prior to baseline (Month 0), and the post-treatment annual eGFR slope was calculated as the change from baseline to Month 6, annualized to a 12-month rate. Within-group longitudinal changes - including pre-finerenone versus post-finerenone eGFR slopes (overall, by eGFR categories, and by dose/TWA subgroups), as well as changes in UACR, UPCR, serum potassium, and blood pressure - were evaluated using the paired Student's t-test or the Wilcoxon signed-rank test, as appropriate. The magnitude of slope improvement (Δslope = post-finerenone slope - pre-finerenone slope) served as the primary dependent variable. To identify independent baseline clinical factors contributing to Δslope improvement, multivariable linear regression analysis was performed. A two-sided p-value of < 0.05 was considered statistically significant.

## Results

Baseline characteristics

Table 1 shows the baseline characteristics of the subjects. The mean age was 68.7 years, and the mean baseline eGFR was 47.5 mL/min/1.73 m2. According to baseline eGFR categories, 17.3% of patients were classified as eGFR ≥ 60 (G1-G2), 37.5% as 45 to < 60 (G3a), 37.5% as 30 to < 45 (G3b), and 7.7% as < 30 mL/min/1.73 m2 (G4). At baseline, the median UACR was 131.4 mg/gCr (IQR: 15.9-500.9 mg/gCr; available in n = 21), and the median UPCR was 1.18 g/gCr (IQR: 0.55-1.90 g/gCr; available in n = 35). The mean serum potassium level was 4.29 mmol/L. Concomitant medications included renin-angiotensin system (RAS) inhibitors in 68.3%, SGLT2 inhibitors in 63.5%, and GLP-1 receptor agonists in 31.7% of patients.

**Table 1:** Baseline clinical characteristics of the study participants (n = 104). Data are presented as mean ± standard deviation, median [IQR], or number (%) as appropriate. Data were available for 21 patients for UACR and 35 patients for UPCR due to the retrospective nature of the study. eGFR, estimated glomerular filtration rate; K, potassium; HbA1c, glycated hemoglobin; LDL, low-density lipoprotein; HDL, high-density lipoprotein; UACR, urinary albumin-to-creatinine ratio; UPCR, urinary protein-to-creatinine ratio; RAS, renin-angiotensin system; ARB, angiotensin II receptor blocker; ACE, angiotensin-converting enzyme; SGLT2, sodium-glucose cotransporter 2; GLP-1, glucagon-like peptide-1; DPP-4, dipeptidyl peptidase-4; IQR, interquartile range.

Parameter	All Patients (n = 104)
Sex	
Male, n (%)	72 (69.2)
Female, n (%)	32 (30.8)
Age (years)	68.7 ± 9.8
Height (m)	1.63 ± 0.10
Body weight (kg)	69.8 ± 14.6
Body mass index (kg/m²)	26.2 ± 5.0
Baseline eGFR (mL/min/1.73 m²)	47.5 ± 15.7
Baseline eGFR categories (CKD stages)	
≥ 60 mL/min/1.73 m² (G1–G2), n (%)	18 (17.3)
45 to < 60 mL/min/1.73 m² (G3a), n (%)	39 (37.5)
30 to < 45 mL/min/1.73 m² (G3b), n (%)	39 (37.5)
< 30 mL/min/1.73 m² (G4), n (%)	8 (7.7)
Serum potassium (mmol/L)	4.29 ± 0.41
Systolic blood pressure (mmHg)	134.6 ± 17.5
Diastolic blood pressure (mmHg)	75.5 ± 10.9
HbA1c (%)	7.14 ± 0.92
LDL-cholesterol (mg/dL)	102.6 ± 24.7
HDL-cholesterol (mg/dL)	57.6 ± 13.9
Triglycerides (mg/dL)	152.7 ± 92.1
UACR (mg/gCr), median [IQR]	131.4 [15.9–500.9]
UPCR (g/gCr), median [IQR]	1.18 [0.55–1.90]
Concomitant medications, n (%)	
RAS inhibitors	71 (68.3)
ARBs	69 (66.3)
ACE inhibitors	2 (1.9)
Diuretics	13 (12.5)
β-blockers	18 (17.3)
SGLT2 inhibitors	66 (63.5)
GLP-1 receptor agonists	33 (31.7)
DPP-4 inhibitors	48 (46.2)
Statins	70 (67.3)

Longitudinal changes in eGFR and eGFR slope

Figure 2 shows the changes in eGFR during the study period. Prior to finerenone initiation, the mean pre-initiation eGFR slope was -5.20 mL/min/1.73 m²/year (95% CI: -7.00 to -3.40). Following finerenone initiation, the mean eGFR slope was +0.27 mL/min/1.73 m²/year (95% CI: -2.42 to +2.96), showing a statistically significant change (p = 0.0144).

**Figure 2:**
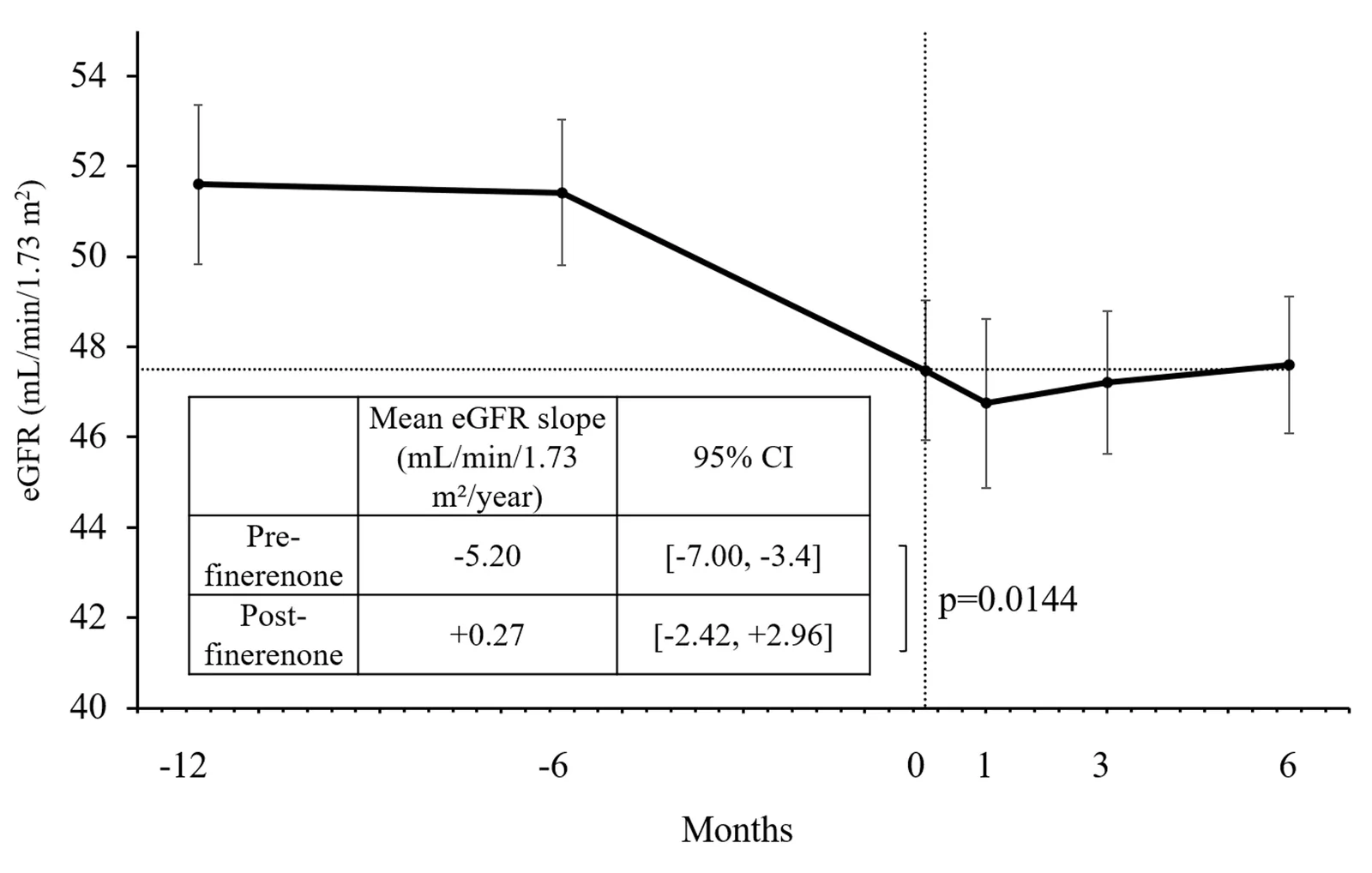
Longitudinal changes in eGFR and mean eGFR slope before and after finerenone initiation. Changes in eGFR and mean eGFR slope in patients with diabetic kidney disease followed for 6 months after finerenone initiation (n = 104). Mean eGFR declined at a rate of -5.20 mL/min/1.73 m^2^/year (95% CI: -7.00 to -3.40) during the one-year pre-finerenone period and significantly reversed to +0.27 mL/min/1.73 m^2^/year (95% CI: -2.42 to +2.96) during the six-month post-finerenone period (Wilcoxon signed-rank test, W = 3453.5, Z = 2.45, p = 0.0144). Data are expressed as mean eGFR slope with 95% CIs. Statistical significance between pre- and post-initiation slopes was evaluated using the Wilcoxon signed-rank test. eGFR, estimated glomerular filtration rate; CI, confidence interval.

Estimated glomerular filtration rate (eGFR) slope changes by baseline eGFR categories

Subgroup analyses of eGFR slope changes according to baseline eGFR categories are summarized in Table 2. In the G3b stage (baseline eGFR between 30 and < 45 mL/min/1.73 m², n = 39), the mean eGFR slope changed from -5.62 mL/min/1.73 m²/year (95% CI: -8.96 to -2.28) pre-initiation to +4.15 mL/min/1.73 m²/year (95% CI: 0.41 to 7.90) post-initiation (p = 0.0019). In stages G1+G2, G3a, and G4, the differences between pre- and post-initiation slopes did not reach statistical significance (p = 0.559, p = 0.223, and p = 0.116, respectively).

**Table 2:** Changes in eGFR slope before and after finerenone initiation according to baseline eGFR categories. Data are presented as mean eGFR slope (mL/min/1.73 m²/year) with 95% confidence intervals [95% CI]. Statistical significance between pre- and post-initiation slopes was evaluated using the paired Student's t-test (*p < 0.05). eGFR, estimated glomerular filtration rate; CI, confidence interval.

Baseline eGFR Category (CKD Stage)	n	Mean eGFR Slope Before Finerenone (mL/min/1.73 m²/year) [95% CI]	Mean eGFR Slope After Finerenone (mL/min/1.73 m²/year) [95% CI]	Test Statistic (t)	p-value
G1–G2 (≥ 60 mL/min/1.73 m²)	18	-4.27 [-8.78, 0.25]	-6.90 [-13.88, 0.08]	-0.6	0.559
G3a (45 to < 60 mL/min/1.73 m²)	39	-4.90 [-7.82, -1.99]	-0.43 [-5.55, 4.68]	1.24	0.223
G3b (30 to < 45 mL/min/1.73 m²)	39	-5.62 [-8.96, -2.28]	+4.15 [0.41, 7.90]	3.34	0.0019*
G4 (< 30 mL/min/1.73 m²)	8	-6.72 [-12.24, -1.21]	+0.63 [-6.45, 7.70]	1.79	0.116

Estimated glomerular filtration rate (eGFR) slope changes stratified by maintenance dose and time-weighted average dose

When stratified by the final maintenance dose at six months, both the 10 mg/day and 20 mg/day groups comprised 52 patients each (Table 3). Baseline eGFR was significantly lower in the 10 mg/day group compared with the 20 mg/day group (43.3 ± 13.8 vs. 51.6 ± 16.7 mL/min/1.73 m²; p = 0.0070; Table 3). Regarding renal function trajectory, patients maintained on 10 mg/day showed a marked improvement in the mean eGFR slope from -7.11 mL/min/1.73 m²/year (95% CI: -9.96 to -4.26) pre-initiation to +3.35 mL/min/1.73 m²/year (95% CI: -0.51 to +7.21) post-initiation (paired t-test, t = 3.78, p = 0.00041; Table 4). In contrast, the 20 mg/day group exhibited a stable trajectory without significant slope change (-3.29 mL/min/1.73 m²/year (95% CI: -5.48 to -1.10) pre-initiation vs. -2.85 mL/min/1.73 m²/year (95% CI: -6.57 to +0.87) post-initiation; paired t-test, t = 0.17, p = 0.865; Table 4). In a direct between-group comparison, the improvement in eGFR slope was significantly greater in the 10 mg/day group than in the 20 mg/day group (+10.46 vs. +0.44 mL/min/1.73 m²/year; p = 0.017).

**Table 3:** Baseline clinical characteristics of study participants stratified by final maintenance dose of finerenone. Data are presented as mean ± standard deviation (SD), median [IQR], or number (%). Statistical comparisons between the two groups were performed using Student's t-test (t) for continuous variables, the Mann-Whitney U test (U) for UACR, and Fisher's exact test for categorical variables. Asterisks indicate statistical significance (*p < 0.05). Baseline UACR data were available for a subset of 21 patients (n = 8 in 10 mg group, n = 13 in 20 mg group). eGFR, estimated glomerular filtration rate; UACR, urinary albumin-to-creatinine ratio; SGLT2, sodium-glucose cotransporter 2; SD, standard deviation; IQR, interquartile range.

Parameter	10 mg/day (n = 52)	20 mg/day (n = 52)	Test Statistic	p-value
Age (years)	68.6 ± 8.9	68.8 ± 10.8	t = -0.13	0.898
Baseline eGFR (mL/min/1.73 m²)	43.3 ± 13.8	51.6 ± 16.7	t = -2.75	0.0070*
Serum potassium (mmol/L)	4.33 ± 0.42	4.26 ± 0.40	t = 0.83	0.411
UACR (mg/gCr), median [IQR]	52.4 [5.4–194.4]	358.1 [94.9–1077.1]	U = 30.5	0.128
SGLT2 inhibitor use, n (%)	36 (69.2)	30 (57.7)	Fisher's exact	0.309

**Table 4:** Changes in eGFR slope before and after finerenone initiation stratified by final maintenance dose. Data are presented as mean eGFR slope (mL/min/1.73 m²/year) with 95% confidence intervals [95% CI]. Δslope represents the post-finerenone slope minus the pre-finerenone slope. Statistical comparisons between pre- and post-initiation slopes were performed using the paired Student's t-test (*p < 0.05). eGFR, estimated glomerular filtration rate; CI, confidence interval.

Maintenance Dose Group	n	Mean eGFR Slope Before Finerenone (mL/min/1.73 m²/year) [95% CI]	Mean eGFR Slope After Finerenone (mL/min/1.73 m²/year) [95% CI]	Δslope (mL/min/1.73 m²/year)	Test Statistic (t)	p-value
10 mg/day	52	-7.11 [-9.96, -4.26]	+3.35 [-0.51, +7.21]	10.46	3.78	0.00041*
20 mg/day	52	-3.29 [-5.48, -1.10]	-2.85 [-6.57, +0.87]	0.44	0.17	0.865

Similarly, when evaluated by time-weighted average (TWA) dose, baseline clinical characteristics showed a lower baseline eGFR in the TWA < 15 mg/day group (n = 61) compared with the TWA ≥ 15 mg/day group (n = 43) (44.1 ± 14.1 vs. 52.3 ± 17.0 mL/min/1.73 m²; p = 0.0085; Table 5). Longitudinal slope analysis demonstrated a significant slope recovery in the TWA < 15 mg/day group, improving from -6.88 mL/min/1.73 m²/year (95% CI: -9.49 to -4.27) to +2.50 mL/min/1.73 m²/year (95% CI: -1.07 to +6.06) (paired t-test, t = 3.65, p = 0.0005; Table 6). Conversely, the slope in the TWA ≥ 15 mg/day group remained unchanged (-2.82 mL/min/1.73 m²/year (95% CI: -5.05 to -0.59) vs. -2.94 mL/min/1.73 m²/year (95% CI: -7.07 to +1.19); paired t-test, t = -0.04, p = 0.967; Table 6).

**Table 5:** Baseline clinical characteristics of study participants stratified by time-weighted average (TWA) dose of finerenone. Data are presented as mean ± standard deviation (SD), median [IQR], or number (%). Statistical comparisons between the two groups were performed using Student's t-test (t) for continuous variables, the Mann-Whitney U test (U) for UACR, and Fisher's exact test for categorical variables. Asterisks indicate statistical significance (*p < 0.05). Baseline UACR data were available for a subset of 21 patients (n = 10 in TWA < 15 mg/day group, n = 11 in TWA ≥ 15 mg/day group). TWA, time-weighted average; eGFR, estimated glomerular filtration rate; UACR, urinary albumin-to-creatinine ratio; SGLT2, sodium-glucose cotransporter 2; SD, standard deviation; IQR, interquartile range.

Parameter	TWA < 15 mg/day (n = 61)	TWA ≥ 15 mg/day (n = 43)	Test Statistic	p-value
Age (years)	69.2 ± 9.0	68.0 ± 11.0	t = 0.59	0.56
Baseline eGFR (mL/min/1.73 m²)	44.1 ± 14.1	52.3 ± 17.0	t = -2.69	0.0085*
Serum potassium (mmol/L)	4.31 ± 0.41	4.26 ± 0.41	t = 0.63	0.533
UACR (mg/gCr), median [IQR]	52.4 [5.8–320.4]	358.1 [97.5–789.0]	U = 33.0	0.13
SGLT2 inhibitor use, n (%)	41 (67.2)	25 (58.1)	Fisher's exact	0.41

**Table 6:** Changes in eGFR slope before and after finerenone initiation stratified by time-weighted average (TWA) dose. Data are presented as mean eGFR slope (mL/min/1.73 m²/year) with 95% confidence intervals [95% CI]. Δslope represents the post-finerenone slope minus the pre-finerenone slope. Statistical comparisons between pre- and post-initiation slopes were performed using the paired Student's t-test (*p < 0.05). TWA, time-weighted average; eGFR, estimated glomerular filtration rate; CI, confidence interval.

TWA Dose Category	n	Mean eGFR Slope Before Finerenone (mL/min/1.73 m²/year) [95% CI]	Mean eGFR Slope After Finerenone (mL/min/1.73 m²/year) [95% CI]	Δslope (mL/min/1.73 m²/year) [95% CI]	Test Statistic (t)	p-value
TWA < 15 mg/day	61	-6.88 [-9.49, -4.27]	+2.50 [-1.07, +6.06]	+9.38 [4.24, 14.51]	3.65	0.0005*
TWA ≥ 15 mg/day	43	-2.82 [-5.05, -0.59]	-2.94 [-7.07, +1.19]	-0.12 [-5.84, 5.60]	-0.04	0.967

Predictors of eGFR slope improvement

Multivariable linear regression analysis was performed to identify baseline factors associated with eGFR slope improvement (Δslope) (Table 7). Concomitant SGLT2 inhibitor use was independently associated with a greater increase in Δslope (B = +9.79, 95% CI: 1.84 to 17.74; p = 0.016). In patients receiving SGLT2 inhibitors, the mean eGFR slope changed from -7.30 mL/min/1.73 m²/year pre-initiation to +3.00 mL/min/1.73 m²/year post-initiation. In addition, younger age (B = -0.58, p = 0.004), lower baseline eGFR (B = -0.28, p = 0.023), and lower body mass index (BMI; B = -0.84, p = 0.025) were independently associated with Δslope improvement. Sex, GLP-1 receptor agonist use, and RAS inhibitor use were not significantly associated with Δslope.

**Table 7:** Multivariable linear regression analysis of baseline factors associated with eGFR slope improvement (Δslope) (n = 104). Δslope was defined as the post-finerenone eGFR slope minus the pre-finerenone eGFR slope (mL/min/1.73 m²/year). Multivariable linear regression analysis was performed using baseline clinical variables (overall model statistics: R² = 0.218, Adjusted R² = 0.161, F (7, 96) = 3.82, p = 0.001). Asterisks indicate statistical significance (*p < 0.05, **p < 0.01). B, unstandardized regression coefficient; CI, confidence interval; eGFR, estimated glomerular filtration rate; SGLT2, sodium-glucose cotransporter 2; GLP-1, glucagon-like peptide-1; RAS, renin-angiotensin system; BMI, body mass index.

Variable	Unstandardized Coefficient (B)	95% Confidence Interval [95% CI]	t-statistic	p-value
SGLT2 inhibitor use (Yes vs. No)	9.79	1.84 to 17.74	2.44	0.016*
Age (per 1-year increase)	-0.58	-0.97 to -0.19	-2.76	0.004**
Baseline eGFR (per 1 mL/min/1.73 m² increase)	-0.28	-0.53 to -0.04	-2.2	0.023*
BMI (per 1 kg/m² increase)	-0.84	-1.57 to -0.11	-2.23	0.025*
GLP-1 receptor agonist use (Yes vs. No)	-2.85	-11.08 to 5.39	-0.32	0.494
Sex (Male vs. Female)	-1.26	-9.12 to 6.59	-0.38	0.75
RAS inhibitor use (Yes vs. No)	0.8	-6.96 to 8.56	0.22	0.838

Changes in UACR and UPCR

Figure 3 and Figure 4 show the changes in UACR and UPCR after finerenone initiation. In the subgroup of 10 patients with paired UACR data, a reduction was observed in nine patients, and the overall decrease was statistically significant (p = 0.0098). Among 30 patients with paired UPCR data, the median UPCR changed from 1.36 g/gCr at baseline to 1.15 g/gCr post-initiation, which did not reach statistical significance (p = 0.839).

**Figure 3:**
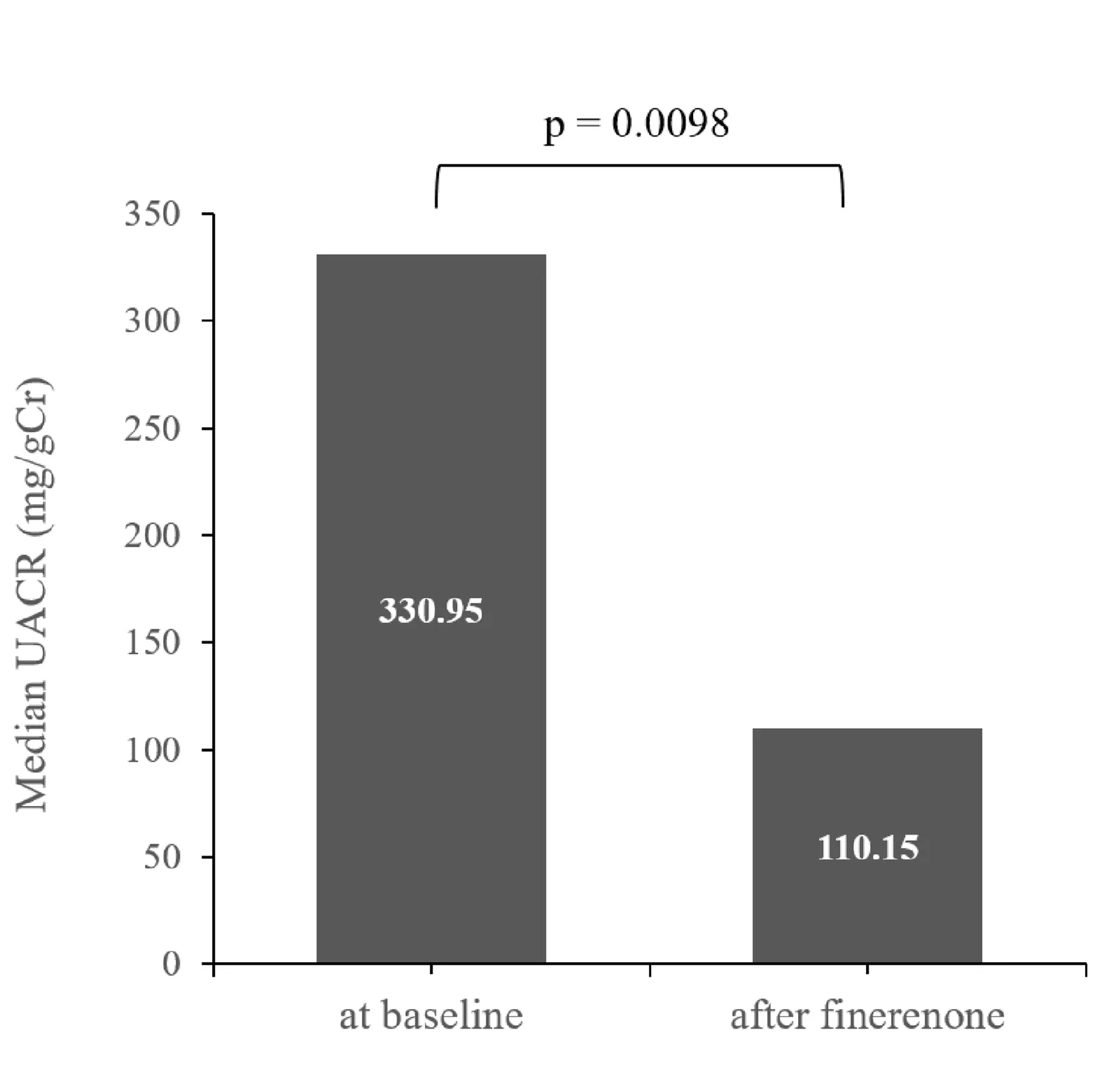
Longitudinal changes in UACR before and after finerenone initiation. Longitudinal changes in UACR from baseline to 6 months post-finerenone initiation in patients with available paired data (n = 21). UACR significantly decreased following finerenone treatment (Wilcoxon signed-rank test, W = 184.0, p = 0.0098). Data are shown as median. UACR, urinary albumin-to-creatinine ratio.

**Figure 4:**
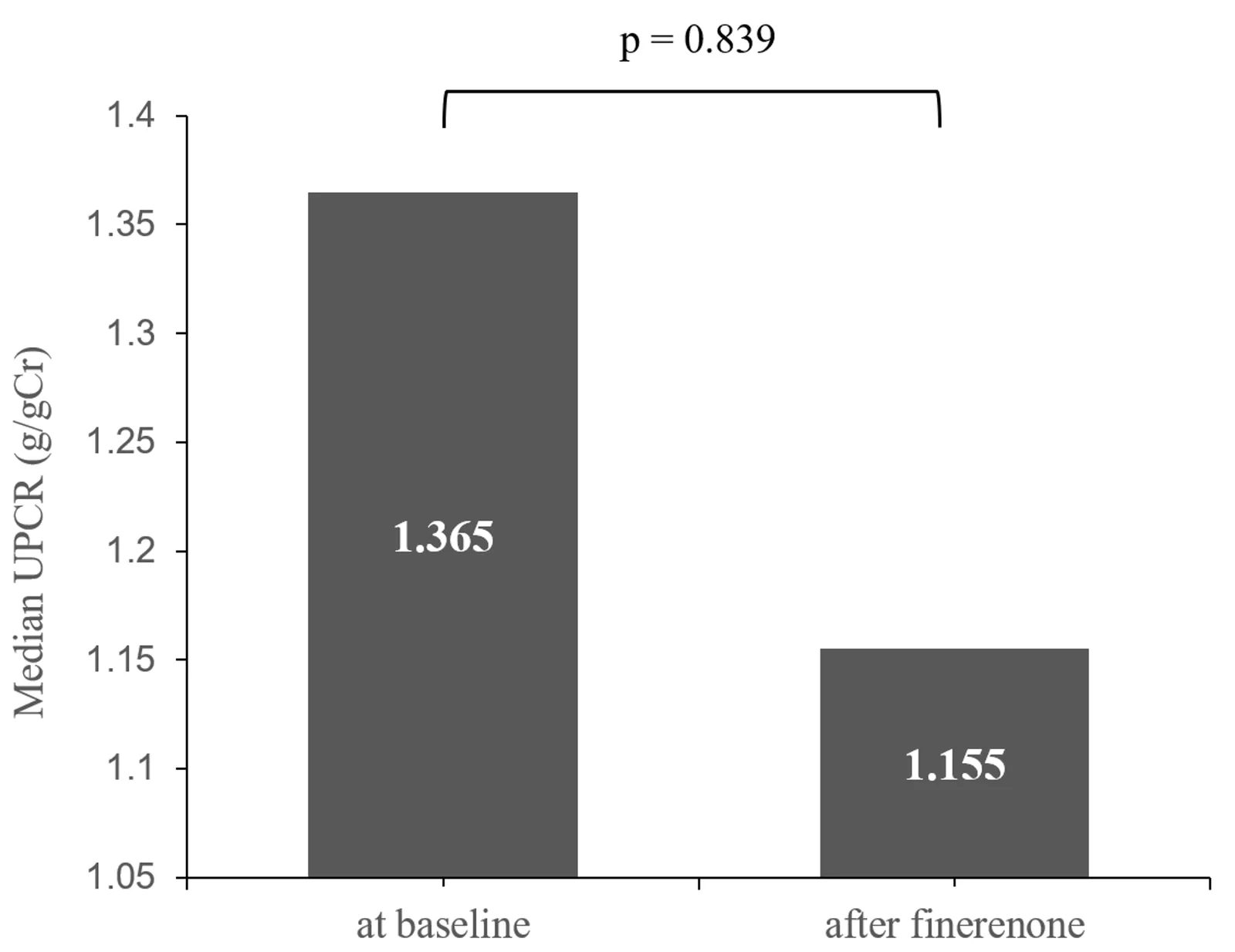
Longitudinal changes in UPCR before and after finerenone initiation. Longitudinal changes in UPCR from baseline to 6 months post-finerenone initiation in patients with available paired data (n = 30). No statistically significant change was observed (Wilcoxon signed-rank test, W = 217.5, p = 0.839). Data are shown as median. UPCR, urinary protein-to-creatinine ratio.

Safety and tolerability

Figure 5 shows the longitudinal changes in serum potassium levels. Serum potassium levels increased from 4.29 ± 0.41 mmol/L at baseline to 4.36 ± 0.45 mmol/L at six months (p = 0.045). Regarding blood pressure, the mean change from baseline to six months was -1.41 mmHg (95% CI: -5.07 to +2.24 mmHg; p = 0.445) for systolic blood pressure and -1.59 mmHg (95% CI: -3.63 to +0.45 mmHg; p = 0.126) for diastolic blood pressure (Figure 6).

**Figure 5:**
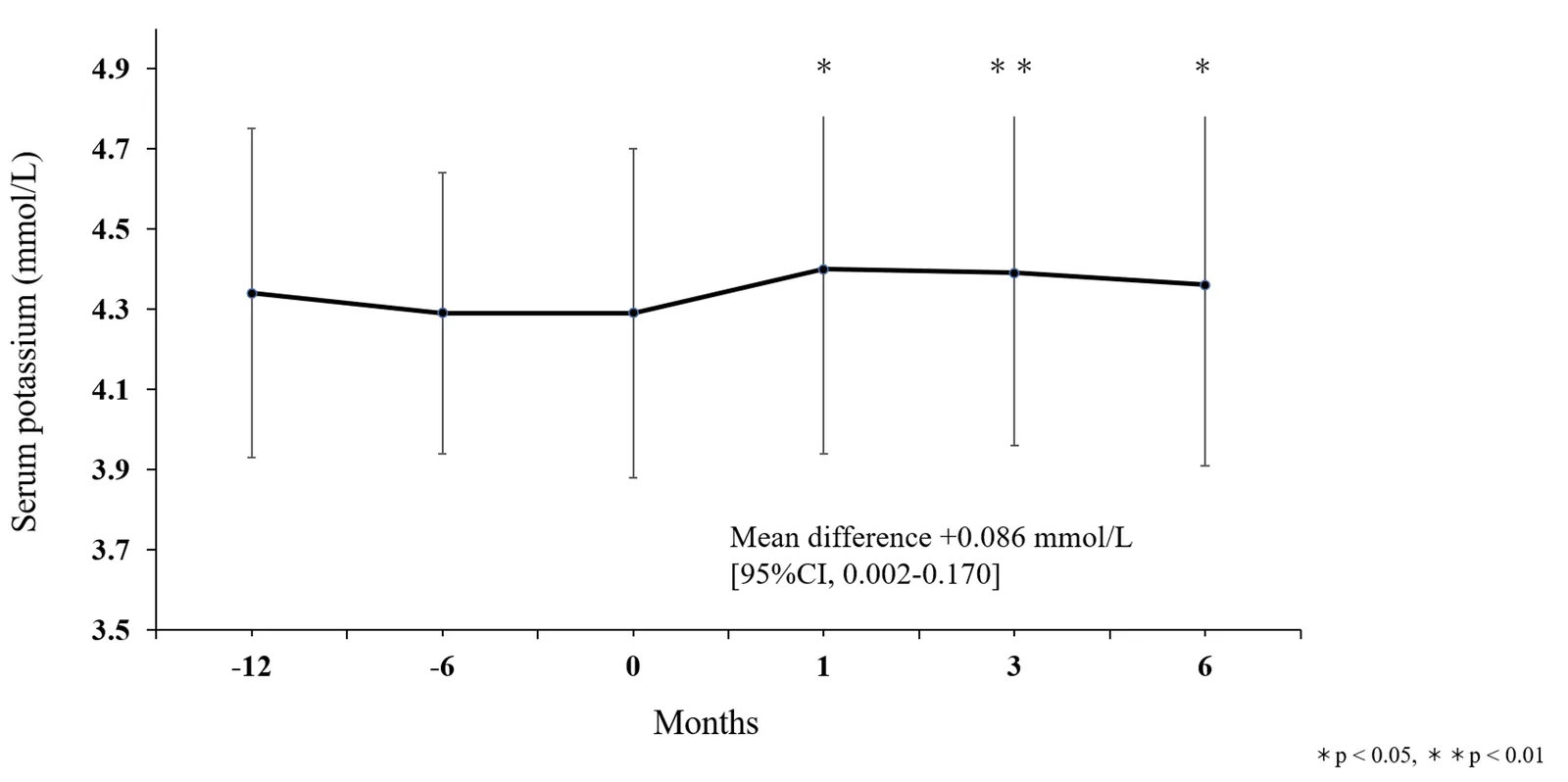
Longitudinal changes in serum potassium levels before and after finerenone initiation. Serum potassium levels at baseline (Month 0; 4.29 ± 0.41 mmol/L), 1 month, 3 months, and 6 months post-initiation (4.41 ± 0.45 mmol/L) in the overall cohort (n = 104). All reported p-values and statistical comparisons represent longitudinal changes with respect to baseline (Month 0) evaluated using the paired Student's t-test (baseline vs. 6 months: t = 2.03, p = 0.045). Data are presented as mean ± standard deviation (SD).

**Figure 6:**
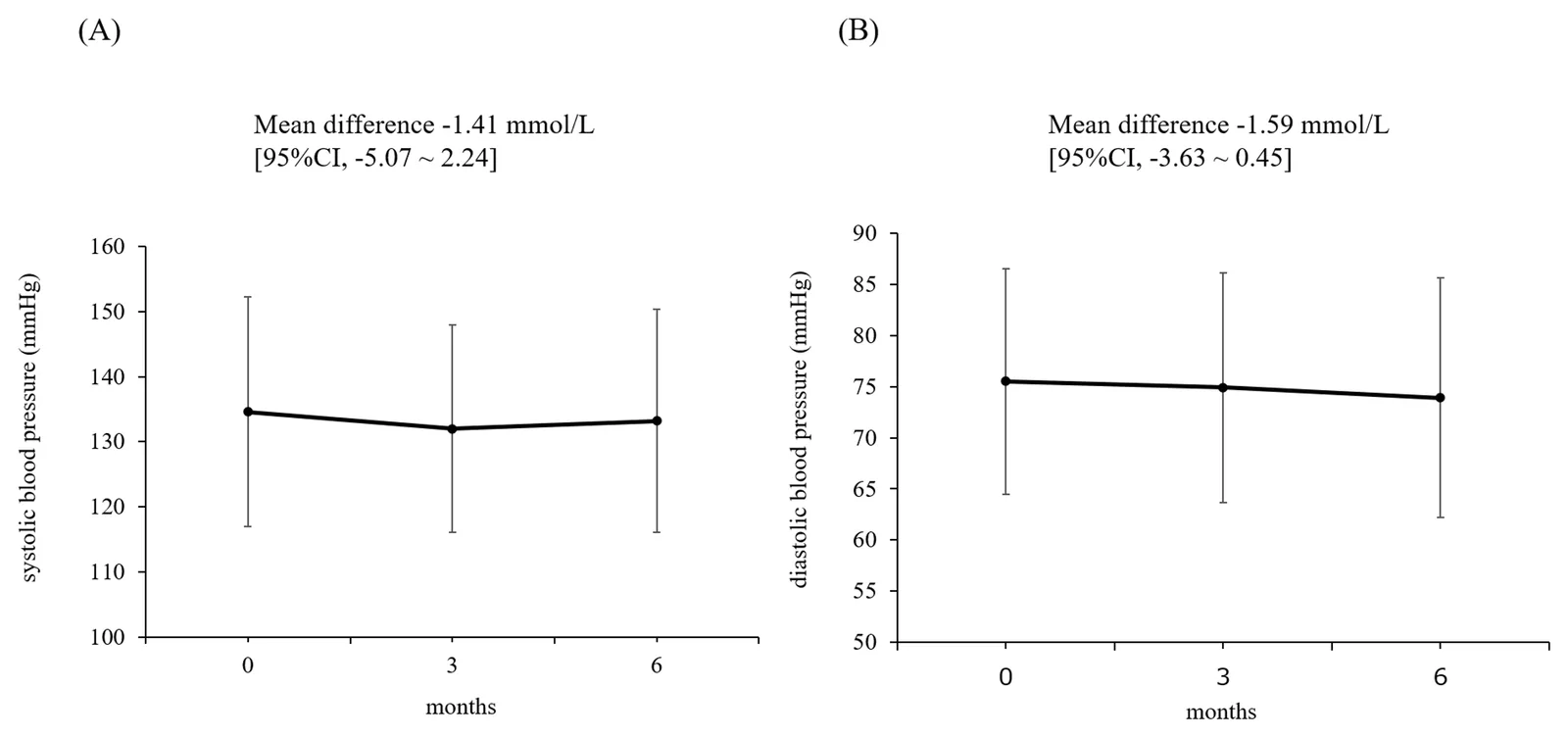
Longitudinal changes in blood pressure before and after finerenone initiation. Changes in (A) systolic blood pressure and (B) diastolic blood pressure from baseline (Month 0) to 6 months following finerenone initiation (n = 104). The mean change from baseline to 6 months was -1.41 mmHg (95% CI: -5.07 to +2.24 mmHg; paired Student's t-test, t = -0.77, p = 0.445) for systolic blood pressure and -1.59 mmHg (95% CI: -3.63 to +0.45 mmHg; paired Student's t-test, t = -1.54, p = 0.126) for diastolic blood pressure. No significant blood pressure fluctuations or excess risk of clinical hypotension were observed over the six-month period. Data are presented as mean change with 95% CIs. Statistical comparisons were performed using the paired Student's t-test. CI, confidence interval.

## Discussion

In this real-world retrospective observational study of Japanese patients with T2DM and DKD, finerenone initiation significantly attenuated the rate of eGFR decline, with the overall mean eGFR slope improving from -5.20 to +0.27 mL/min/1.73 m²/year (p = 0.0144). Crucially, our findings offer two key clinical insights: first, maintaining finerenone at a lower dose of 10 mg/day conferred substantial eGFR slope improvement in patients presenting with rapid baseline decline; second, concomitant SGLT2 inhibitor use was independently associated with a greater increase in eGFR slope. These observations align with accumulating real-world evidence demonstrating the effectiveness of finerenone across diverse clinical phenotypes, including advanced kidney dysfunction and nephrotic conditions [14,15]. 

The renoprotective benefits observed in real-world settings are grounded in finerenone’s distinct pharmacological and molecular profile [4-6]. Unlike traditional steroidal MRAs, finerenone is a bulky, nonsteroidal antagonist that exhibits structural inverse agonism at the mineralocorticoid receptor (MR) [4]. By competitively preventing cofactor recruitment without inducing the distinct conformational shifts typical of classical MR agonists, finerenone potently suppresses downstream pro-inflammatory signaling pathways, including nuclear factor-κB (NF-κB) transactivation and the overexpression of downstream chemokines (e.g.,** **monocyte chemoattractant protein-1 (MCP-1)/C-C motif chemokine ligand 2 (CCL2))** **and cytokines (e.g., interleukin 6 (IL-6) and tumor necrosis factor alpha (TNF-α)) [4-6]. Concurrently, finerenone disrupts pro-fibrotic signaling cascades, attenuating transforming growth factor-β1 (TGF-β1) and connective tissue growth factor (CTGF) expression, thereby mitigating pathological extracellular matrix accumulation and preserving renal parenchymal integrity [4,6]. Furthermore, by blunting MR-mediated nicotinamide adenine dinucleotide phosphate (​​​​​​​NADPH)oxidase upregulation, finerenone reduces renal oxidative stress, restores endothelial nitric oxide bioavailability, and alleviates microvascular endothelial injury [4].

In stage-stratified analysis, significant slope improvement was evident in patients with moderately reduced kidney function (CKD stage G3b, -5.62 to +4.15 mL/min/1.73 m²/year). Although statistical significance was not reached in the preserved renal function cohort (CKD stages G1-G2), this was likely attributable to the small subgroup size (n = 18) and the physiological initial dip in intraglomerular pressure, which can mask chronic slope stabilization during a short six-month observation window [7,16].

Regarding dosage, patients maintained on 10 mg/day exhibited marked slope recovery (-7.11 to +3.35 mL/min/1.73 m²/year), whereas those on 20 mg/day remained in a stable trajectory (-3.29 to -2.85 mL/min/1.73 m²/year). This divergence reflects an inherent indication bias typical of real-world practice, where attending physicians cautiously maintain lower doses in patients with more advanced renal impairment or higher hyperkalemia risk (Tables 3, 5). Although the direct between-group comparison showed a statistically greater slope improvement in the 10 mg/day group, this does not imply the superiority of a specific dose. Instead, it demonstrates that even when uptitration to the standard 20 mg/day dose is clinically precluded, maintaining finerenone at 10 mg/day provides meaningful therapeutic benefit in attenuating rapid kidney function loss.

Multivariable linear regression identified concomitant SGLT2 inhibitor use as a strong independent predictor of slope improvement (+9.79 mL/min/1.73 m²/year). This synergistic benefit likely arises from complementary intrarenal hemodynamics: afferent arteriolar constriction induced by SGLT2 inhibitors combined with efferent vasodilation by finerenone, which cooperatively alleviates intraglomerular hypertension [17,18]. While younger age, lower baseline eGFR, and lower body mass index were also associated with greater slope gains, glucagon-like peptide-1 receptor agonist and renin-angiotensin system inhibitor use were not independent predictors in this acute timeframe. Unlike the rapid hemodynamic action of SGLT2 inhibitors, these agents primarily exert slower, structural renoprotective effects that typically manifest over years [19,20], which were unlikely to be captured within our six-month follow-up.

Regarding safety, finerenone significantly reduced urinary albumin-to-creatinine ratio (p = 0.0098) without predisposing patients to excess hyperkalemia or clinical hypotension, confirming its clinical tolerability.

Translating these molecular actions into routine clinical practice presents distinct practical challenges. First, clinicians must differentiate the acute initial eGFR dip (a transient hemodynamic reduction within the first few weeks) from subsequent chronic renal preservation [7,16]. This dip reflects the therapeutic reduction of intraglomerular hypertension via efferent arteriolar vasodilation; recognizing this physiological adaptation is crucial to avoid unwarranted, premature drug discontinuation [7,14]. Second, despite clinical trial protocols recommending uptitration to 20 mg/day [7], real-world evidence indicates that a substantial proportion of patients remain maintained on the lower 10 mg/day dose, largely reflecting clinical caution regarding hyperkalemia and renal impairment in routine practice [10,11]. Third, navigating polypharmacy complexities - specifically, sequencing and combining finerenone with SGLT2 inhibitors and RAS inhibitors - requires close vigilance over volume status and serum potassium to optimize hemodynamic synergy [3,17]. Finally, notable clinical limitations include practical constraints in advanced renal impairment (e.g., baseline eGFR < 25 mL/min/1.73 m²), where safety margins narrow and real-world evidence remains evolving [14,15], as well as the uncertain transitional impact in patients with prior exposure to steroidal MRAs [12].

Several limitations must be acknowledged. First, this was a single-center retrospective study lacking an untreated control group; thus, non-pharmacological confounders, such as regular follow-up visits or lifestyle modifications, cannot be entirely excluded. Second, dose allocation was non-randomized and subject to physician discretion and indication bias, precluding definitive conclusions regarding dose superiority. Furthermore, patients maintained on 10 mg/day presented with lower baseline eGFR and steeper pre-treatment decline, which may reflect potential regression to the mean contributing to slope recovery. Although restricting the longitudinal analysis to patients with six-month data warrants consideration, no patients discontinued finerenone due to adverse events, hyperkalemia, or progression to end-stage kidney disease during the observation period. Third, the six-month follow-up period reflects acute hemodynamic adjustments and initial dip dynamics rather than long-term hard renal outcomes. Fourth, unmeasured clinical confounders - including diabetes duration, baseline glycemic control (HbA1c), baseline blood pressure, and heart failure comorbidity - could not be fully adjusted for in our multivariable models. Fifth, paired longitudinal albuminuria data were available in only a subset of patients due to routine clinical constraints. Finally, mathematical coupling between baseline trajectory and change in slope (Δslope) warrants consideration. Nevertheless, our study provides valuable real-world evidence supporting the feasibility and practical dose management of finerenone in clinical practice.

## Conclusions

In conclusion, finerenone significantly attenuated eGFR decline in real-world patients with DKD, with slope improvement observed in patients maintained on the 10 mg/day dose. Concomitant SGLT2 inhibitor use was independently associated with greater improvement in eGFR slope, supporting the clinical utility and practical dose management of finerenone in routine practice.
